# COVID-19 Modifications for Remote Teleassessment and Teletraining of a Complementary Alternative Medicine Intervention for People With Multiple Sclerosis: Protocol for a Randomized Controlled Trial

**DOI:** 10.2196/18415

**Published:** 2020-07-03

**Authors:** Byron Lai, Chia-Ying Chiu, Emily Pounds, Tracy Tracy, Tapan Mehta, Hui-Ju Young, Emily Riser, James Rimmer

**Affiliations:** 1 Division of Pediatric Rehabilitation Medicine School of Medicine University of Alabama at Birmingham Birmingham, AL United States; 2 Department of Health Services Administration School of Health Professions University of Alabama at Birmingham Birmingham, AL United States; 3 Dean's Office School of Health Professions University of Alabama at Birmingham Birmingham, AL United States; 4 Tanner Foundation Birmingham, AL United States; 5 Department of Physical Therapy School of Health Professions University of Alabama at Birmingham Birmingham, AL United States

**Keywords:** multiple sclerosis, telerehabilitation, teletraining, physical activity, disability, tele-exercise, telehealth, COVID-19

## Abstract

**Background:**

Access to comprehensive exercise and rehabilitation services for people with multiple sclerosis (MS) remains a major challenge, especially in rural, low-income areas. Hence, the Tele-Exercise and Multiple Sclerosis (TEAMS) study aims to provide patient-centered, coordinated care by implementing a 12-week complementary and alternative medicine (CAM) intervention for adults with MS. However, due to the societal impact of coronavirus disease (COVID-19) in mid-March 2020, the University of Alabama at Birmingham announced a limited business model halting all nonessential research requiring on-site visits, which includes the TEAMS study.

**Objective:**

In compliance with the shelter-in-place policy and quarantine guidance, a modified testing and training protocol was developed to allow participants to continue the study.

**Methods:**

The modified protocol, which replaces on-site data collection and training procedures, includes a teleassessment package (computer tablet, blood pressure cuff, hand dynamometer, mini disc cone, measuring tape, an 8” step, and a large-print 8” × 11” paper with ruler metrics and wall-safe tape) and a virtual meeting platform for synchronous interactive training between the therapist and the participant. The teleassessment measures include resting blood pressure and heart rate, grip strength, Five Times Sit to Stand, Timed Up & Go, and the Berg Balance Scale. The teletraining component includes 20 sessions of synchronous training sessions of dual tasking, yoga, and Pilates exercises designed and customized for a range of functional levels. Teletraining lasts 12 weeks and participants are instructed to continue exercising for a posttraining period of 9 months.

**Results:**

The protocol modifications were supported with supplemental funding (from the Patient-Centered Outcomes Research Institute) and approved by the University Institutional Review Board for Human Use. At the time nonessential research visits were halted by the university, there were 759 people enrolled and baseline tested, accounting for 92.5% of our baseline testing completion target (N=820). Specifically, 325 participants completed the 12-week intervention and follow-up testing visits, and 289 participants needed to complete either the intervention or follow-up assessments. A modified analysis plan will include sensitivity analyses to ensure the robustness of the study results in the presence of uncertainty and protocol deviations. Study results are projected to be published in 2021.

**Conclusions:**

This modified remote teleassessment/teletraining protocol will impact a large number of participants with MS who would otherwise have been discontinued from the study.

**Trial Registration:**

ClinicalTrials.gov NCT03117881; https://clinicaltrials.gov/ct2/show/NCT03117881

**International Registered Report Identifier (IRRID):**

DERR1-10.2196/18415

## Introduction

Multiple sclerosis (MS) is an autoimmune-mediated neurological disorder that results in demyelination and transection of axons in the central nervous system, and it affects more than two million people across the world [1,2]. The current literature strongly supports the use of home telerehabilitation or what is commonly referred to as telehealth (to encompass a broader set of uses) as an equally effective alternative to usual care for patients with MS [3-6]. Because exercise has a strong effect in managing health and MS symptoms [7-10], exercise rehabilitation is one of the major applications of telehealth technology. The advantages of telehealth over usual care include increased social support, participant adherence, quality of care, cost-effectiveness, access to services (no need for transportation), and reduced burden on health professionals to allow easier dissemination of services [11]. Since comprehensive exercise and rehabilitation services are not provided on-site across all MS clinics, remote testing and training can make such services more accessible for patients who do not live near center-based programs.

In 2017, our research team began to implement a randomized controlled effectiveness trial comparing two methods of delivering a complementary and alternative medicine (CAM) intervention: CAM delivered at home and on-site at a clinic (the TEAMS [Tele-Exercise and Multiple Sclerosis] study; ClinicalTrials.gov NCT03117881) [12]. The CAM intervention consists of yoga, Pilates, and dual tasking exercises. Participants are randomized into one of two groups: (a) home-based CAM involving the use of a computer tablet with an app to stream 20 prerecorded exercise or rehabilitation video sessions tailored to an individual’s functional level [13]; and (b) clinic-based CAM involving 20 supervised exercise training sessions with trained study therapists. The supervised sessions were implemented by 86 therapists at 43 clinics that were spread across three states (Alabama, Mississippi, and Tennessee). The study originally aimed to include 820 people with MS by April 30, 2020.

In response to coronavirus disease (COVID-19), the University of Alabama at Birmingham halted all nonessential research studies that required on-site interaction with research participants. This included the TEAMS study, which had a total of 759 participants enrolled and baseline tested at the time of closure (March 2020); this accounts for 92.5% of our baseline testing target (N=820). Specifically, 325 participants completed the study, and 289 participants were in the process of completing the study (ie, they needed to either complete the intervention or their follow-up data collection visits at 3-, 6-, and 12-months postintervention). Breaking this sample down further, there were 545 remaining data collection (on-site) visits that needed to be completed at the time of the COVID-19 closure. With no apparent end date to COVID-19, the research team requested a modification to the protocol from the study program officer. The teleassessment and training protocol modifications were approved and supported with supplemental funding provided by the funding agency (Patient-Centered Outcomes Research Institute [PCORI]) on March 27, 2020, and the protocol was approved by the University Institutional Review Board for Human Use on March 29, 2020. This paper describes the COVID-19 protocol modifications.

## Methods

The TEAMS study is a randomized controlled trial comparing the effectiveness of a 12-week tele-exercise program delivered at home via a computer tablet (TeleCAM) or at the clinic by a therapist (DirectCAM). Further details of the TEAMS study can be found elsewhere [12].

### Recruitment

A total of 289 participants are in the process of completing the TEAMS study and will be reconsented to undergo the COVID-19 modified study procedures. Inclusion and exclusion criteria remain the same as the original study (Textbox 1).

**Inclusion and exclusion criteria.**

**Inclusion criteria**

- Patient Determined Disease Steps score: 0-7 (mild-to-moderate disability)
- Able to use arms and/or legs for exercise while standing or seated
- Aged 18-70 years
- Physician permission for study participation

**Exclusion criteria**

- Significant visual acuity that prevents seeing a tablet screen in order to follow along with the home exercise program (self-reported)
- Event(s) of cardiovascular disease, severe pulmonary disease, or renal failure within the last 6 months
- Active pressure ulcer
- Pregnancy
- Received a rehabilitation session within the last 30 days
- Godin Leisure-Time Exercise Questionnaire score ≥24 (ie, physically active)

### The Intervention and COVID-19 Modifications

**Figure 1:**
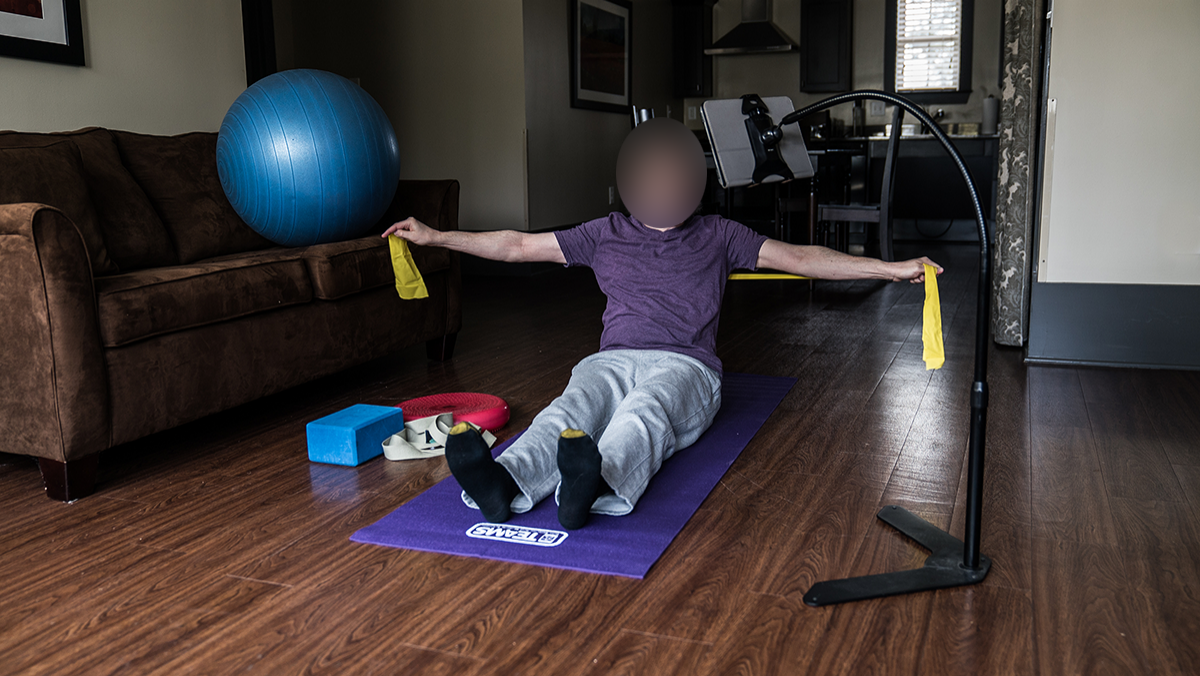
An example of a person with multiple sclerosis exercising in their home with the intervention equipment and computer tablet.

The 12-week DirectCAM intervention is composed of dual tasking, yoga, and Pilates. Participants in DirectCAM receive in-person instruction from a therapist 2 times a week for the first 8 weeks and then once a week for the last 4 weeks for a total of 20 sessions (1 hr/session). TeleCAM participants receive the same 12-week intervention through exercise videos that are preloaded to a custom-designed tablet app [13]. Figure 1 demonstrates a person with MS performing the intervention using the provided equipment and computer tablet. They also receive phone calls through an Interactive Voice Response system to collect data and enhance adherence to the intervention. After the 12-week intervention, DirectCAM participants receive written instructions and photos of the exercises and are encouraged to continue the program at home for 9-months postintervention. TeleCAM participants are encouraged to continue to exercise using the videos on their tablet 9-months postintervention. Currently, there is a total of 545 participants for whom data collection needs to be completed: 88 participants that need to complete follow-up data collection at 3 months postintervention; 168 at 6 months; and 289 at 12 months. There are 25 participants who were randomized to receive DirectCAM but were unable to because of COVID-19 restrictions.

In response to COVID-19, PCORI provided the study team with supplemental funding, which was used to adapt the on-site intervention into remote testing and training with a licensed occupational or physical therapist. DirectCAM participants are now receiving their remaining intervention sessions with a study therapist through videoconferencing. These participants are categorized into a separate group referred to as remote DirectCAM (rDirectCAM). rDirectCAM participants who decline or are unable to videoconference (eg, no compatible computer or internet access at home) have the option of communicating with their therapist via telephone. Thus, this study includes two methods of teletraining: (1) internet-supervised training (rDirectCAM) and (2) self-regulated training using preloaded videos on a computer tablet (TeleCAM).

### Teleassessments

A total of 16 therapists will conduct all assessments through videoconferencing (teleassessments). To ensure strong fidelity across therapists, they will undergo a 30-minute training session directed by the clinical study coordinator (author TT). TT is a licensed occupational therapist with 24 years of training and has been involved with the TEAMS study since its inception. TT will also train and monitor therapists to deliver rDirectCAM through videoconferencing.

#### A Brief Description of Assessment Measures and Protocols

##### Participants Who Decline or Are Unable to Participate in Teleassessments

Participants who decline or are unable to participate in the teleassessments will be sent the study questionnaires either through mail or email via the study database (Research Electronic Data CAPture [REDCap]), based on their preference. Questionnaires include the 36-Item Short Form Survey (SF-36), the Modified Fatigue Impact Scale, and the Godin Leisure Time Exercise Questionnaire (for more details refer to our original study protocol [12]).

##### Protocol for Teleassessment Participants

**Figure 2:**
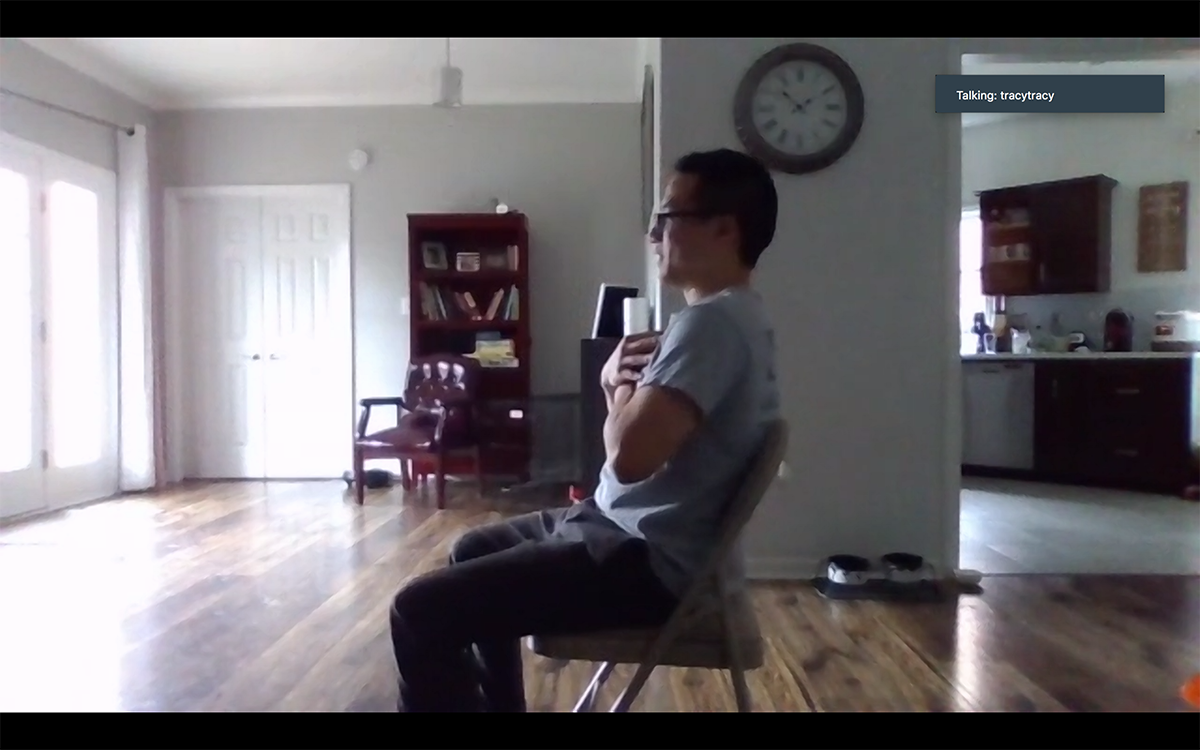
An example of the videoconference view of the starting position for the Five Times Sit to Stand test.

**Figure 3:**
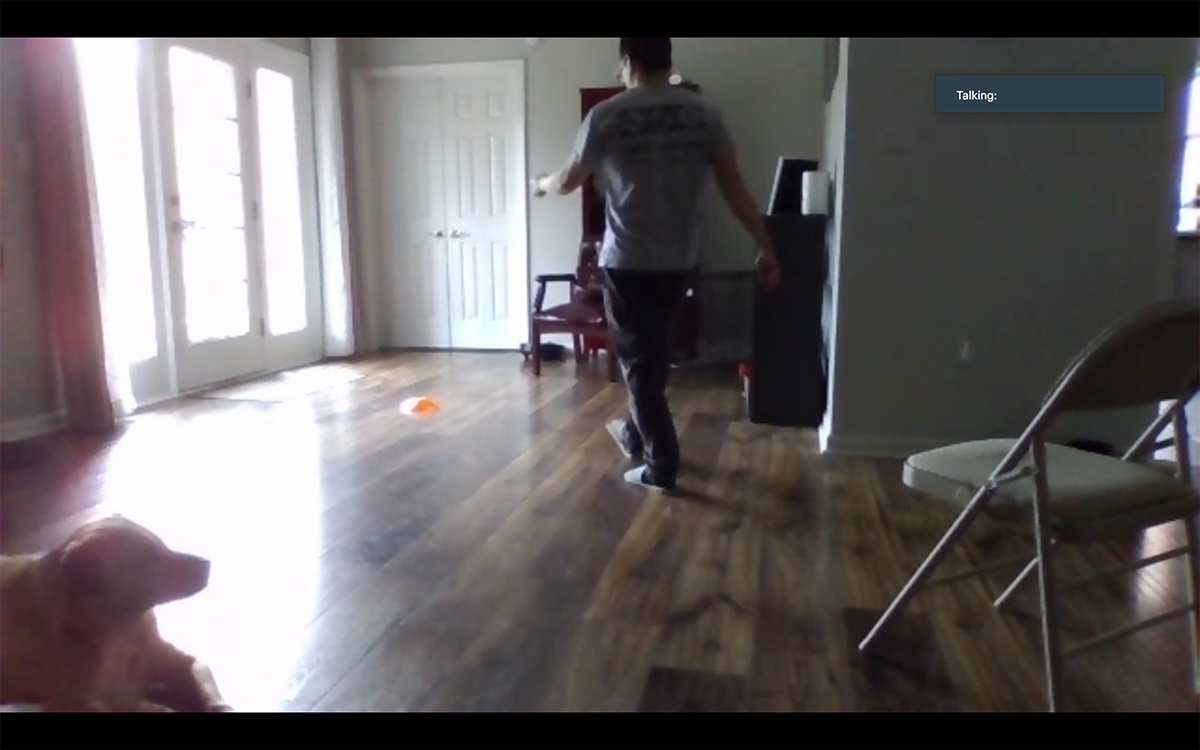
An example of the videoconference view for the Timed Up & Go test.

**Figure 4:**
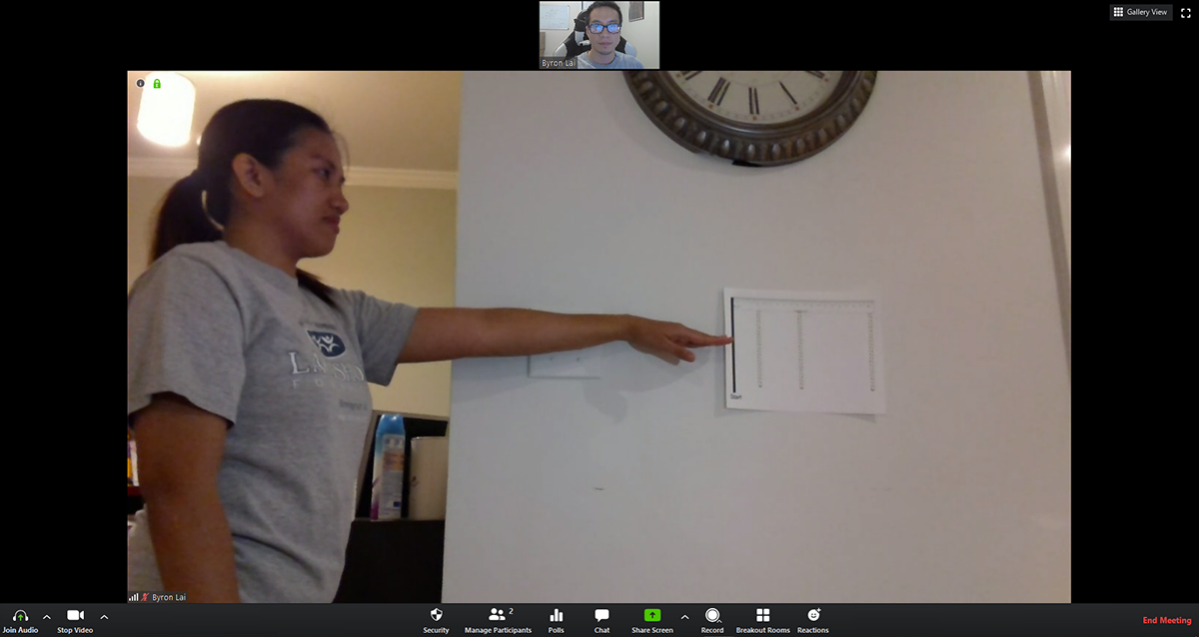
An example of the videoconference view for the starting position of the functional reach task of the Berg Balance Scale.

The goal of the teleassessment protocol is to mirror the on-site assessment protocol [12]. The measures have strong psychometric properties to support their use in people with MS. The measures that participants undergo depend on their Patient Determined Disease Steps (PDDS) score. Participants with a PDDS score between 0-4 (indicative of minimal mobility disability) will complete all measures: the Hand-Grip Strength Test [14], the Five Times Sit to Stand test [15], the Timed Up & Go Test [16-18], and the Berg Balance Scale [19,20]. Participants with a PDDS score between 5-7 complete all measures except the Timed Up & Go Test and Berg Balance Scale (due to safety concerns as noted previously during on-site clinic visits). The teleassessment measures are listed below, and specific details and procedures can be found in the supplemental data collection form and therapist guide (Multimedia Appendices 1 and 2, respectively):

- Height and weight (self-report)
- Changes in medication (self-report)
- Blood pressure/heart rate
  Equipment: a chair with an arm, a heart rate monitor, and a blood pressure cuff
  Setup:
    Camera view: frontal view of the participant’s upper body
    The participant sits quietly for at least 5 minutes prior to assessment
- Hand-grip strength
  Equipment: a chair with an arm and a digital hand dynamometer
  Setup:
    Camera view: frontal view of the participant’s upper body
    The participant sits in a stationary chair or a wheelchair and uses a hand dynamometer; three trials with each hand with the elbow flexed at 90 degrees, with 30-second rest in between trials
- Five Times Sit to Stand (Figure 2)
  Equipment: a chair (a chair with arms can enhance safety but discourage the participant from using, if unnecessary)
  Setup:
    Camera view: side view of the participant’s entire body (at least shoulders, hip, and knees)
    The participant sits in a chair or a wheelchair
    Three options for where to place the chair:
      An open space, not supported by a mat or wall
      An open space, support from a caregiver or family member, if necessary
      Supported against a wall (if chair movement is an issue)
- Timed Up & Go (Figure 3)
  Conducted only for people with PDDS Score between 0-4, determined via self-report during prescreening.
  Equipment: a chair with arms, a mini disc cone, and a 3-meter soft measuring tape
  Setup:
    Camera view: diagonal or frontal view to capture the participant’s entire body throughout the test (camera view should include the 3-meter walkway and the chair)
    The participant can place the computer tablet on the floor or on furniture for better view
    The participant places a chair at the beginning of a 12-foot cleared space, free from obstacles or throw rugs
    The participant lays down the measuring tape starting at the tip of their toes when sitting in a chair and places the cone at the end of the measuring tape
    The participant removes the measuring tape
    Chair setup options:
      An open space, not supported by a mat or wall
      An open space, support from a caregiver/or family member, if needed
      Supported against a wall
- Berg Balance Scale (note: the research team anticipated that the Berg Balance Scale would be the most difficult test to administer via telehealth. Thus, the team incorporated elements from a previous study demonstrating that the Berg could be conducted reliably through teleassessment [21])
  Conducted only for people with PDDS Score between 0-4, determined via self-report during prescreening.
  Equipment: two chairs (one with arms and one without), an 8” therapy step, a printed paper with ruler metrics and wall safe tape, and a mini disc cone
  Setup:
    Camera view includes the frontal view of the participant’s entire body for all tasks
    Three options for where to place the chair:
      An open space, not supported by a mat or wall
      An open space, support from a caregiver/or family member, if possible
      Supported against a wall
  Test instructions and modifications:
    All tasks completed in frontal view
    After completing all tasks once, repeat the unsupported standing and standing with eyes closed task with a side view (in order to reduce the likelihood of missing data) [21]
    Perform the functional reach task with a custom print paper with large-numbered metrics and markings to indicate critical scoring criteria for the therapist (Figure 4).

#### Teleassessment Equipment

**Figure 5:**
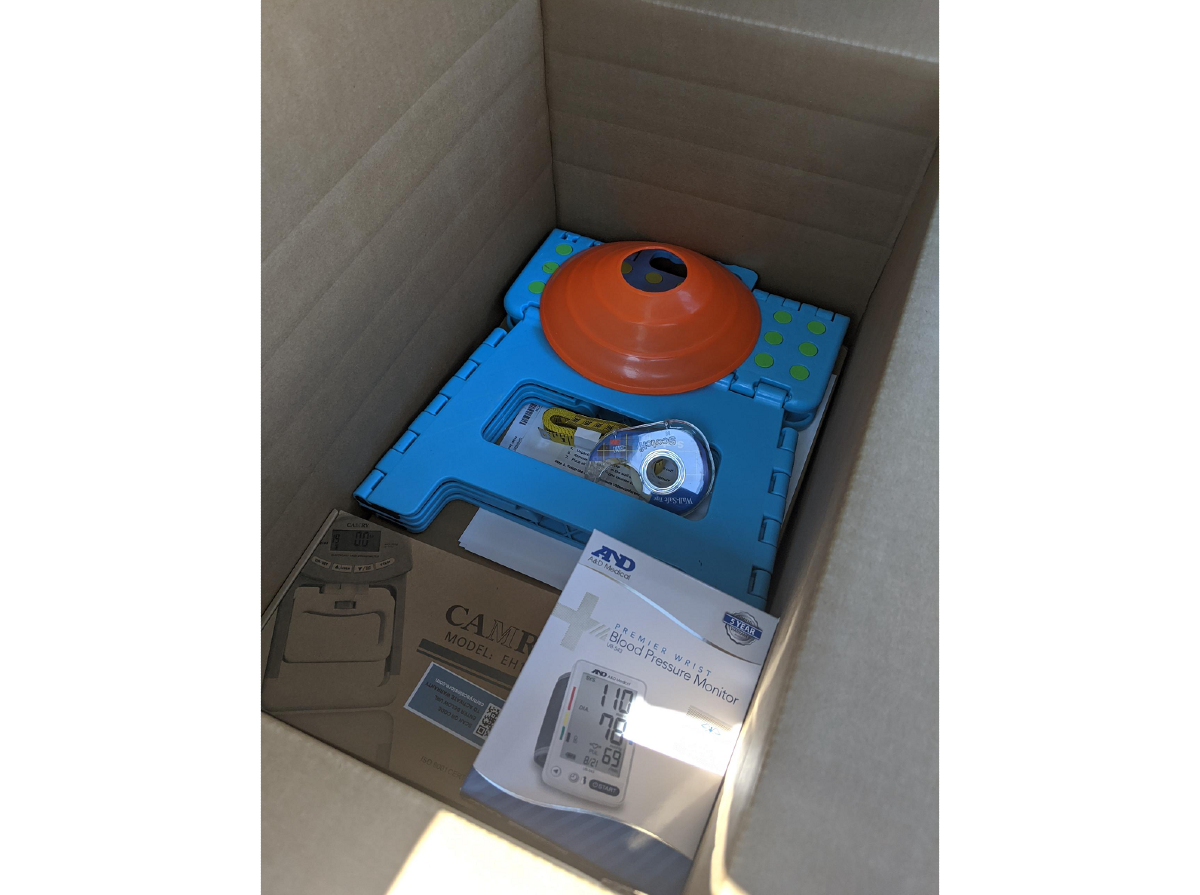
A teleassessment package prior to being shipped to a participant's home. The equipment includes an 8" step, a blood pressure cuff, a roll of wall-safe tape, a 3-meter measuring tape, a hand dynamometer, a mini disc cone, a large print paper with ruler metrics, a sheet with participant instructions (under the step), and a computer laptop (bottom).

After participants have reconsented, they will be asked whether they would like to participate in the teleassessments. Research personnel will ship interested participants a single package (minimum package dimensions 18.5” × 12.1” × 8.75”) containing the following teleassessment equipment (Figure 5):

- A roll of wall-safe tape;
- Computer tablet (Lenovo S340 81TB0000US);
- Heart rate and blood pressure cuff (A&D Medical Wrist Blood Pressure Monitor, UB-543);
- CAMRY Hand Grip Dynamometer;
- American Challenge Soccer Mini Disc Cones (required to complete the Timed Up & Go test and used for the pick-up task of the Berg Balance Scale);
- Soft measuring tape, precut to a 3-meter length for the Timed Up & Go test;
- An 8” small foldable step stool (required to complete the Berg Balance Scale);
- A large-print 8” × 11” paper with ruler metrics.

#### General Teleassessment Procedures

Teleassessments will be conducted at baseline and at 3, 6, and 12 months postintervention. Given the size of the project and the strong psychometric properties of the teleassessments, the research team was primarily concerned with whether the teleassessments could be delivered reliably across different therapists. Accordingly, the first 8 participants who will receive the teleassessments will participate in a preliminary interrater reliability study. These 8 participants will complete 2 teleassessment sessions (done in the same day). Each teleassessment session will be conducted by one of two therapists. A sample size of 8 was chosen based on an intraclass correlation (ICC) power calculation with the following components: statistical power of 0.8; α=0.05; two raters; minimum acceptable reliability (H0)=0.75, expected reliability (H1)=0.97. The value for expected reliability was based on a systematic review paper of interrater reliability of the Berg Balance Scale in a clinical population [22]. We anticipated that the Berg Balance Scale would be the most difficult test to conduct via teleassessment.

Therapists will schedule the teleassessments with study participants and send them a Zoom meeting link that can be accessed via the provided tablet. During the meeting, therapists will have access to two documents to help guide them through the teleassessments: (1) a manual for quick visual reminders, and (2) a step-by-step data collection form to be completed during the meeting (Multimedia Appendix 2). Therapist interactions with the participant will be guided by the Supportive Accountability Theory [23]. Specifically, therapists will aim to develop a social relationship/bond and present a social presence that provides a sense of accountability to enhance participants’ motivation and adherence to the teleassessment protocol. The procedures for the teleassessments are listed in Textbox 2.

**General teleassessment procedures.**

1. Open data collection form in the box
2. Start videoconference meeting with the participant
3. Build social bond with the participant (introductions and establish rapport)
4. Brief the participant on what the teleassessment will entail and duration (max 2 hours)
5. Ask the participant if their medication has changed and document additions
6. Ask the participant if they have all the necessary equipment and sufficient space
7. Assess the room for safety hazards or obstacles
8. Ensure the participant has adequate privacy
9. Inform the participant to be careful during teleassessments
10. Complete anthropometrics and functional assessments using the data collection form
11. Remind the participant to complete the questionnaires that were sent to them
12. Inform the participant that research personnel will send them a gift card
13. Schedule the next teleassessment follow-up (if applicable)
14. End the videoconference meeting
15. Upload the data collection forms to Box and notify research personnel that testing is complete and to send a gift card

### Statistical Analysis

All statistical analyses for the TEAMS study intervention will be conducted in an intent-to-treat manner at the individual level considering the multilevel and repeated nature of the data (participants nested within clinics with 4 time points for every participant). We will conduct analyses with an additional time-varying covariate to account for protocol deviations (rDirectCAM and participants who received teleassessments). Contrasts and estimate statements will be used to draw inferences pooling estimates from the original intervention types (DirectCAM versus TeleCAM). We will conduct sensitivity analyses with and without the rDirectCAM participants. Given that the rDirectCAM group was created unexpectedly in response to COVID-19, this group may not be adequately powered to detect changes in outcomes.

Regarding the teleassessment interrater reliability examination, ICC values of 0.5-0.75 will be interpreted as moderate reliability; 0.75-0.9 good reliability; and 0.9 excellent reliability [24]. We will also collect feasibility data related to participant uptake and implementation processes throughout the remainder of the TEAMS study and publish this in a future paper. These data will include the (1) number of participants who volunteer for the teleassessments; (2) number of participants who complete the follow-up teleassessments at months 3, 6, and 12 postintervention; (3) number of teleassessment tasks completed; (4) total time in minutes required to complete the teleassessments; and (5) adverse events.

## Results

**Figure 6:**
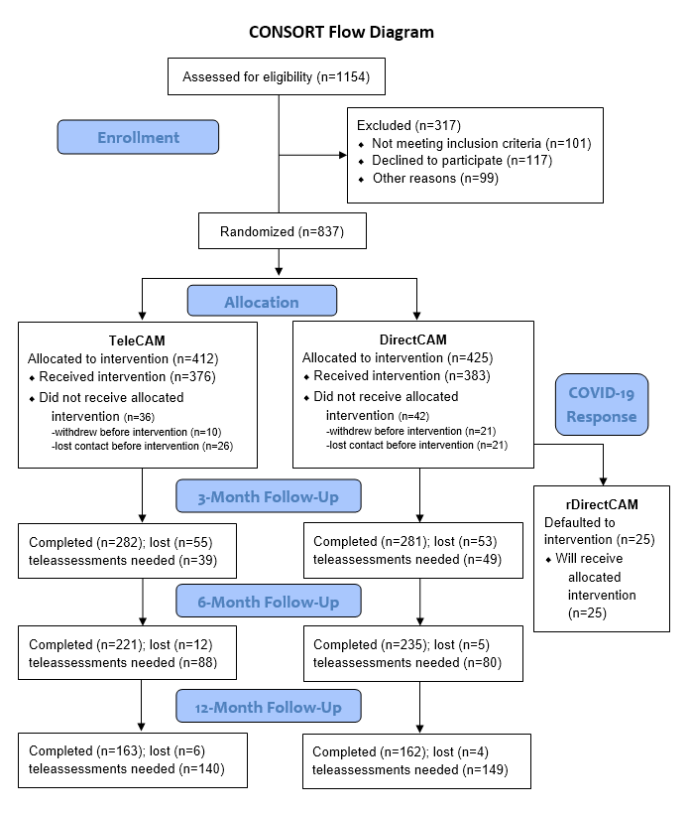
Modified CONSORT (Consolidated Standards of Reporting Trials) diagram reporting the number of teleassessments that need to be completed and participants who need to undergo rDirectCAM. COVID-19: coronavirus disease.

Recruitment commenced on March 16, 2020, using a multipronged recruitment strategy. Participants were recruited via study brochures or word of mouth from MS clinics and specialists, a large three-state (Alabama, Mississippi, and Tennessee) community organization network, and through the networks of a diverse MS stakeholder team that was engaged in the project [12]. Recruitment is now complete and there are 837 individuals with MS randomized into the study. Data collection is ongoing. Details on data collected for participant characteristics (eg, type of MS, disability severity, and functional mobility) and outcomes are reported elsewhere [12]. The modified CONSORT (Consolidated Standards of Reporting Trials) diagram (Figure 6) displays consented, enrolled, and completed participants, as well as the number of teleassessments required to complete the study.

## Discussion

This paper describes a modified teleassessment and teletraining protocol for the TEAMS intervention due to COVID-19 restrictions on on-site visits. To the best of our knowledge, this study will also include the largest number of teleassessments ever conducted in a telerehabilitation/exercise trial for people with MS.

The study findings will be of great relevance to health professionals who aim to conduct similar remote, synchronous telerehabilitation/tele-exercise trials for people with MS and other disability groups. While the research team matched as closely as possible the standardized on-site assessment procedures with the remote teleassessment protocol, the psychometric properties (ie, validity and reliability) of the specific teleassessment procedures have not yet been tested. We aim to establish the psychometric properties of these tests once COVID-19 restrictions are lifted. General feasibility data will be recorded for all participants who undergo the teleassessments (eg, participants volunteering for the teleassessments, percentage of tasks completed/not completed, and time to implement the tests). These findings will be used to support evidence-based policy decisions regarding telerehabilitation implementation, development, and programming.

## Appendix

Multimedia Appendix 1 Teleassessment data collection form.

Multimedia Appendix 2 Therapist instruction guide.

## Abbreviations

CAM: complementary alternative medicine
CONSORT: Consolidated Standards of Reporting Trials
COVID-19: coronavirus disease
ICC: intraclass correlation
MS: multiple sclerosis
PCORI: Patient-Centered Outcomes Research Institute
PDDS: Patient Determined Disease Steps
RCT: randomized controlled trial
REDCap: Research Electronic Data CAPture
SF-36: 36-Item Short Form Survey
TEAMS: Tele-Exercise And Multiple Sclerosis
