# Supplementary material for: COVID-19 Modifications for Remote Teleassessment and Teletraining of a Complementary Alternative Medicine Intervention for People With Multiple Sclerosis: Protocol for a Randomized Controlled Trial
Source: JMIR Res Protoc. 2020 Jul 3;9(7):e18415. doi: 10.2196/18415 (PMC7337611; doi:10.2196/18415)
Supplement: Multimedia Appendix 1 [file resprot_v9i7e18415_app1.docx]

**Data Collection Form**

(For Tele-Assessment Use)

| **Participant ID:** ____________ | **Visit (circle one): BL FU3 FU6 FU12** |
| --- | --- |
| **PDDS score: ________**  **Staff ID:** ______________ | **Date:** ______________ |

**Any changes to medication list?** 🞏 Yes* 🞏 No

*****If YES please indicate changes here: _______________________________________________

*****Please record all values to one decimal place.*****

**Anthropometrics**

- **Height (Self-Reported)**:
- **Weight (Self-Reported)**:
- **BP/HR**: 1^st^ Reading _____ / ______ Resting HR: _________bpm L / R arm

2^nd^ Reading _____ / ______ Resting HR: _________bpm L / R arm

- **Is BP over 160/100**? 🞏 Yes* 🞏 No
- **Is resting HR over 100 bpm**? 🞏 Yes* 🞏 No

*If YES do NOT test and reschedule visit.

- **Notes**: _________________________________________________________________

**Grip Strength (90**­­**°)**

- **Dominant Hand**: Right / Left

|  | **Trial 1** | **Trial 2** | **Trial 3** |
| --- | --- | --- | --- |
| **Right Hand (lbs)** |  |  |  |
| **Left Hand (lbs)** |  |  |  |

**Notes (e.g. reason for incompletion):** ________________________________________

________________________________________________________________________

**Five Times Sit to Stand Test (FTSST)**

- **Measured chair height: __________cm**
- **Does the chair participant use/have a seat cushion? 🞏Yes 🞏 No**
- **Time to complete test:** __________________ seconds
- **Notes (e.g. reason for incompletion):** ________________________________________

_______________________________________________________________________

**Timed Up and Go (TUG)**

- **View: 🞏 Frontal 🞏 Diagonal**
- **Measured chair height: __________cm**
- **Does the chair participant uses have seat cushion? 🞏Yes 🞏 No**
- **Does participant use an assistive device? 🞏Yes 🞏 No**
  - **If no, perform TUG**
  - **If yes, testing is complete. Skip to page 3 for scheduling next appointment.**
- **Time to complete test (1^st^ trial):** seconds
- **Time to complete test (2^nd^ trial):** seconds
- **Time to complete test (3^rd^ trial):** seconds
- **Notes (e.g. reason for incompletion):** ________________________________________

_______________________________________________________________________

**Berg Balance Scale (BBS)**

| 1. **Sitting to standing** |  | ___________ |
| --- | --- | --- |
| 1. **Standing unsupported** |  | ___________ |
| 1. **Sitting unsupported** |  | ___________ |
| 1. **Standing to sitting** |  | ___________ |
| 1. **Transfers** |  | ___________ |
| 1. **Standing with eyes closed** |  | ___________ |
| 1. **Standing with feet together** |  | ___________ |
| 1. **Reaching forward with outstretched arm** 2. **Retrieving object from the floor** 3. **Turning to look behind** 4. **Turning 360 degrees** 5. **Placing alternate foot on stool** 6. **Standing with one foot in front** 7. **Standing on one foot** |  | ____________  ____________  ____________  ____________  ____________  ____________  ____________ |
| **Total BBS Score** |  | __________ **/ 56** |

- **Notes (e.g. reason for incompletion):** ________________________________________

________________________________________________________________________

**Time to complete the entire tele-assessment (start to end of the call):** __________________ minutes

**Problems/issues during testing (if applicable):** _____________________________________________

_____________________________________________________________________________________

_____________________________________________________________________________________

**Next Visit (circle one and put date): FU3 FU6 FU12 , Date:________________________**

**Testing Instructions**

**Equipment Checklist for Testing Set Up**

Assessor need following list:

🞏 Stopwatch

🞏 Data collection form

🞏 Laptop/computer with video camera

🞏 Headset if needed

🞏 Enough space to demonstrate tests for participant

Participant need following list:

🞏 A delivered tele-assessment equipment package

🞏 A computer laptop

🞏 A HR monitor and BP cuff

🞏 A digital hand dynamometer

🞏 Mini disc cones

🞏 A 118.11-inch (3-meter) soft measuring tape

🞏 An 8-inch step

🞏 Berg paper with measurements and wall tape

🞏 Two chairs (one with arms and one without; Berg Balance Scale Only / PDDS 1-4)

🞏 A 12-foot unobstructed walkway

🞏 A table or piece of furniture to position computer and web camera on to view testing space

## Blood Pressure/Heart Rate

**Set Up:** Have the participant sit in a stationary chair or their wheelchair quietly for at least 5 minutes.

**Participant Instructions:** Measure blood pressure on the left side unless contraindicated. Make sure the legs are uncrossed, feet flat on the floor. Have participant place their arm on a table, pillow, or across their lap so that it is supported at heart level with their palm facing up. Place blood pressure cuff around wrist half an inch above your wrist bone. Push start on the monitor and remain still until the cuff has inflated and deflated.

Ask the participant to show the therapist the BP monitor screen and record the numbers on the excel sheet in box. Do this twice. Record each of these measures on the excel sheet in Box.

**Grip Strength (90°)**

**Set Up:** Have the participant sit in a stationary chair and use a hand dynamometer from the tele-assessment equipment package.

**Participant Instructions:** Ask the participant to “*Sit or transfer into a stationary chair*”. Determine the participant’s hand dominance by asking, *“Are you right-handed or left-handed?”* Document the answer on the data collection sheet. Have the participant sit in the chair, shoulder adducted and neutrally rotated, elbow by their side and flexed to a 90° angle, and forearm and wrist in neutral position. Tell the participant “*Bend your elbow to a 90-degree angle and place the dynamometer in your dominant hand. Press (ON/SET) button to turn the dynamometer on. When you are ready, hit (START) button and squeeze the handle as hard as you can when I say squeeze. You will do this three times on each hand. Now squeeze, then let me (therapist) see the reading on the dynamometer. Please wait 30 seconds, and squeeze again when I give you the command”*  Do this two more times on the dominant hand, then repeat procedure on the non-dominant hand.

Record each of these measures on the excel sheet in Box.

**FTSST**

**Set Up:** Make sure the chair is in an open space, not supported against a mat or wall. You may use a chair with arms for safety but discourage participant from using if unnecessary. If you feel participant needs more support or have safety concerns the participant can: 1) push chair up against a wall 2) place the chair on carpet 3) have a caregiver stand nearby.

A lateral view of the participant works best for this test.

**Participant Instructions:** *“Please sit back against the chair with your arms folded across your chest. I want you to stand up and sit down 5 times as quickly as you can when I say ‘Go’. ‘Go.’”*

Do NOT talk to the participant while he/she is performing the test. The participant may have a practice round or you may demonstrate the test to participant if fatigue is a concern. Participant should be asked to stop if the therapist is concerned for the participant’s safety at any point during the test. Record the duration (in seconds with two decimal places) of the participant completing 5 sit to stands on the data collection form.

**TUG**

**Set Up:** Have the participant place a chair at the beginning of a 12-foot cleared space, free from obstacles or throw rugs. Next, have the participant lay down the measuring tape starting at the tip of their toes (may need to stabilize by putting dynamometer on top of the tape) when sitting in a chair, then place the cone at the end of the measuring tape. Now remove the measuring tape and sit down in the chair.

You will need to see the participant’s chair and the walking path. A diagonal view of the room may work best, but you can also instruct the participant place the laptop on the floor at the end of the walking path to provide you with a frontal view.

**Participant Instructions:** *“Please sit back against the chair. When you are ready, I will say ‘Go’. You will get up from the chair, walk around the cone, and return back to your chair taking a seat.”* Demonstrate for the participant. Begin the watch when you say *“Go”* and stop the watch when the participant’s buttocks touch the seat. Do this procedure two more times.

Participant should be asked to stop if the therapist is concerned for the participant’s safety at any point during the test. Record the duration (in seconds with two decimal places) of each trial from start to finish on the data collection form.

**BBS**

**Set Up:** Ask the participant to take out a therapy step, the minidisc cone, and the Berg measuring paper and an adhesive tape from the delivered package. Have the participant attach the Berg measuring paper to a wall with more than 10” of space. Have the participant get one chair without arms and one chair with arms. Place the step and the minidisc close by these two chairs. If the participant does not have two chairs, a bed may be used.

Please document each task and/or give instructions as written. When scoring, please record the lowest response category that applies for each item. In most items, the subject is asked to maintain a given position for a specific time. Progressively more points are deducted if:

- the time or distance requirements are not met
- the subject’s performance warrants supervision
- the subject touches an external support or receives assistance from the examiner

Subject should understand that they must maintain their balance while attempting the tasks. The choices of which leg to stand on or how far to reach are left to the subject. Poor judgment will adversely influence the performance and the scoring.

Based on therapist clinical judgement, participant may be asked to stand at the corner of a room for standing components of this test for safety precautions. Participant should be asked to stop if the therapist is concerned for the participant’s safety at any point in testing.

The view of the participant in this test is a frontal view. After you have completed the Berg in it’s entirety with the participant, please perform #6 and #7 in a lateral view.

1. SITTING TO STANDING INSTRUCTIONS: Please stand up. Try not to use your hand for support.

- (  )4 able to stand without using hands and stabilize independently
- (  )3 able to stand independently using hands
- (  )2 able to stand using hands after several tries
- (  )1 needs minimal aid to stand or stabilize

( )0 needs moderate or maximal assist to stand

2. STANDING UNSUPPORTED INSTRUCTIONS: Please stand for two minutes without holding on.

( )4 able to stand safely for 2 minutes

( )3 able to stand 2 minutes with supervision

( )2 able to stand 30 seconds unsupported

( )1 needs several tries to stand 30 seconds unsupported

( )0 unable to stand 30 seconds unsupported

If a subject is able to stand 2 minutes unsupported, score full points for sitting unsupported. Proceed to item #4.

3. SITTING WITH BACK UNSUPPORTED BUT FEET SUPPORTED ON FLOOR OR ON A STOOL INSTRUCTIONS: Please sit with arms folded for 2 minutes.

- (  )4 able to sit safely and securely for 2 minutes
- (  )3 able to sit 2 minutes under supervision
- (  )2 able to sit 30 seconds
- (  )1 able to sit 10 seconds
- (  )0 unable to sit without support 10 seconds

4. STANDING TO SITTING INSTRUCTIONS: Please sit down.

- (  )4 sits safely with minimal use of hands
- (  )3 controls descent by using hands
- (  )2 uses back of legs against chair to control descent
- (  )1 sits independently but has uncontrolled descent
- (  )0 needs assist to sit

5. TRANSFERS INSTRUCTIONS: Arrange chair(s) for pivot transfer. Ask subject to transfer one way toward a seat with armrests and one way toward a seat without armrests. You may use two chairs (one with and one without armrests) or a bed and a chair.

- (  )4 able to transfer safely with minor use of hands
- (  )3 able to transfer safely definite need of hands
- (  )2 able to transfer with verbal cuing and/or supervision
- (  )1 needs one person to assist

(  )0 needs two people to assist or supervise to be safe

6. STANDING UNSUPPORTED WITH EYES CLOSED INSTRUCTIONS: Please close your eyes and stand still for 10 seconds.

- (  )4 able to stand 10 seconds safely
- (  )3 able to stand 10 seconds with supervision
- (  )2 able to stand 3 seconds
- (  )1 unable to keep eyes closed 3 seconds but stays safely

(  )0 needs help to keep from falling

7. STANDING UNSUPPORTED WITH FEET TOGETHER INSTRUCTIONS: Place your feet together and stand without holding on.

- (  )4 able to place feet together independently and stand 1 minute safely
- (  )3 able to place feet together independently and stand 1 minute with supervision
- (  )2 able to place feet together independently but unable to hold for 30 seconds
- (  )1 needs help to attain position but able to stand 15 seconds feet together
- (  )0 needs help to attain position and unable to hold for 15 seconds

8. REACHING FORWARD WITH OUTSTRETCHED ARM WHILE STANDING INSTRUCTIONS: Lift arm to 90 degrees. Stretch out your fingers and reach forward as far as you can. (Examiner places a ruler at the end of fingertips when arm is at 90 degrees. Fingers should not touch the ruler while reaching forward. The recorded measure is the distance forward that the fingers reach while the subject is in the most forward lean position. When possible, ask subject to use both arms when reaching to avoid rotation of the trunk.)

- (  )4 can reach forward confidently 25 cm (10 inches)
- (  )3 can reach forward 12 cm (5 inches)
- (  )2 can reach forward 5 cm (2 inches)
- (  )1 reaches forward but needs supervision

(  )0 loses balance while trying/requires external support

9. PICK UP OBJECT FROM THE FLOOR FROM A STANDING POSITION INSTRUCTIONS: Pick up the shoe/slipper, which is in front of your feet.

- (  )4 able to pick up slipper safely and easily
- (  )3 able to pick up slipper but needs supervision

(  )2 unable to pick up but reaches 2-5 cm(1-2 inches) from slipper and keeps balance independently

- (  )1 unable to pick up and needs supervision while trying

(  )0 unable to try/needs assist to keep from losing balance or falling

10. TURNING TO LOOK BEHIND OVER LEFT AND RIGHT SHOULDERS WHILE STANDING INSTRUCTIONS: Turn to look directly behind you over toward the left shoulder. Repeat to the right. (Examiner may pick an object to look at directly behind the subject to encourage a better twist turn.)

- (  )4 looks behind from both sides and weight shifts well
- (  )3 looks behind one side only other side shows less weight shift
- (  )2 turns sideways only but maintains balance
- (  )1 needs supervision when turning
- (  )0 needs assist to keep from losing balance or falling

11. TURN 360 DEGREES INSTRUCTIONS: Turn completely around in a full circle. Pause. Then turn a full circle in the other direction.

- (  )4 able to turn 360 degrees safely in 4 seconds or less
- (  )3 able to turn 360 degrees safely one side only 4 seconds or less
- (  )2 able to turn 360 degrees safely but slowly
- (  )1 needs close supervision or verbal cuing

(  )0 needs assistance while turning

12. PLACE ALTERNATE FOOT ON STEP OR STOOL WHILE STANDING UNSUPPORTED INSTRUCTIONS: Place each foot alternately on the step/stool. Continue until each foot has touched the step/stool four times.

- (  )4 able to stand independently and safely and complete 8 steps in 20 seconds
- (  )3 able to stand independently and complete 8 steps in > 20 seconds
- (  )2 able to complete 4 steps without aid with supervision
- (  )1 able to complete > 2 steps needs minimal assist

(  )0 needs assistance to keep from falling/unable to try

13. STANDING UNSUPPORTED ONE FOOT IN FRONT INSTRUCTIONS: (DEMONSTRATE TO SUBJECT) Place one foot directly in front of the other. If you feel that you cannot place your foot directly in front, try to step far enough ahead that the heel of your forward foot is ahead of the toes of the other foot. (To score 3 points, the length of the step should exceed the length of the other foot and the width of the stance should approximate the subject’s normal stride width.)

- (  )4 able to place foot tandem independently and hold 30 seconds
- (  )3 able to place foot ahead independently and hold 30 seconds
- (  )2 able to take small step independently and hold 30 seconds

(  )1 needs help to step but can hold 15 seconds

(  )0 loses balance while stepping or standing

14. STANDING ON ONE LEG INSTRUCTIONS: Stand on one leg as long as you can without holding on.

- (  )4 able to lift leg independently and hold > 10 seconds
- (  )3 able to lift leg independently and hold 5-10 seconds
- (  )2 able to lift leg independently and hold ≥ 3 seconds
- (  )1 tries to lift leg unable to hold 3 seconds but remains standing independently

( )0 unable to try of needs assist to prevent fall

***REPEAT TESTS BELOW WITH SIDE VIEW (Use Best From Frontal or Side View)***

6. STANDING UNSUPPORTED WITH EYES CLOSED INSTRUCTIONS: Please close your eyes and stand still for 10 seconds.

- (  )4 able to stand 10 seconds safely
- (  )3 able to stand 10 seconds with supervision
- (  )2 able to stand 3 seconds
- (  )1 unable to keep eyes closed 3 seconds but stays safely

(  )0 needs help to keep from falling

7. STANDING UNSUPPORTED WITH FEET TOGETHER INSTRUCTIONS: Place your feet together and stand without holding on.

- (  )4 able to place feet together independently and stand 1 minute safely
- (  )3 able to place feet together independently and stand 1 minute with supervision
- (  )2 able to place feet together independently but unable to hold for 30 seconds
- (  )1 needs help to attain position but able to stand 15 seconds feet together
- (  )0 needs help to attain position and unable to hold for 15 seconds
