# Supplementary material for: COVID-19 Modifications for Remote Teleassessment and Teletraining of a Complementary Alternative Medicine Intervention for People With Multiple Sclerosis: Protocol for a Randomized Controlled Trial
Source: JMIR Res Protoc. 2020 Jul 3;9(7):e18415. doi: 10.2196/18415 (PMC7337611; doi:10.2196/18415)
Supplement: Multimedia Appendix 2 [file resprot_v9i7e18415_app2.docx]

***TEAMS Teleassesssment Guide***

**There are 3 general steps:**

1. Setup (Tele-space and Data Collection Form)
2. Functional Assessments
3. Tracking (Notifying research personnel)

# Procedures

1.
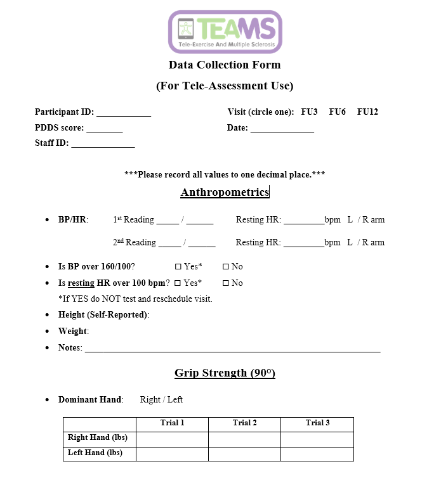
**Open the data collection form in UAB Box Folder** provided to you by research
2. **Host Zoom chat meeting**

(login to Zoom, host a new meeting with the same meeting ID that was sent to the participant, enable the participant into the meeting room from the wait room)

1. **Introduce yourself to the participant**

(try to build a social bond/relationship with the participant, as it will help their responsiveness to the protocol [Supportive Accountability Theory, shown below]). You can ask questions like: How was your day? Did anything interesting happen this week? Tell me one fun fact about yourself? What do you like to do for fun?


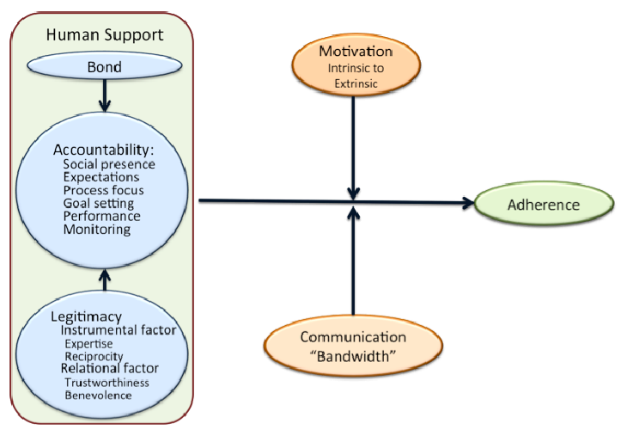


1. **Brief participant on what the teleassesssment will entail and how long it will take (max duration of 2 hours)**
   1. Anthropometric and vital signs measures
   2. Hand-grip strength test
   3. Five Times Sit to Stand Test (FTSST)
   4. Timed Up and Go Test (TUG) (PDDS 1-4 ONLY)
   5. Berg Balance Scale (BBS) (PDDS 1-4 ONLY)
2. **Ask participant if their medication has changed and document additions**
3. **Ask participant if they have all the necessary equipment and space for the teleassesssment**

🞏 A HR monitor and BP cuff

🞏 A digital hand dynamometer

🞏 Mini disc cones

🞏 A 120-inch soft measuring tape

🞏 An 8-inch therapy step

🞏 A 36-inch adhesive measuring ruler

🞏 A 12-foot unobstructed walkway (TUG)

🞏 A table or piece of furniture to position computer and web camera on to view testing space

🞏 Two chairs (BBS / PDDS 1-4; one with arms and one without)

Assessor need following list:

🞏 Stopwatch

🞏 Data collection form

🞏 Laptop/computer with video camera

🞏 Headset if needed

🞏 Enough space to demonstrate tests for participant

1. **Assess the teleassesssment room for safety hazards/obstacles**
   1. Tell participant to move the laptop to show you the four corners of the room
      1. Look for items below and ask participant to address the items if possible, and DO NOT CONDUCT walking tests near obstacles on the floor that could lead to a fall
         1. Physical objects
         2. Uneven surfaces
         3. Changes in surface textures (carpet to hardwood)
2. **Ensure participant has adequate privacy**
   1. Ask participant if they are comfortable with the view of their home/space)
   2. Ensure no identifying information is viewable through the camera (e.g., names, addresses, financial info, etc.)
3. **Inform participant to be careful during the assessments**
   1. Ask participant to fasten seatbelt if using a wheelchair
   2. Ask participant to keep the wheel brakes for wheelchairs or walkers locked until necessary to move
   3. Tell participant to be cautious of their balance when doing the tests and to inform you if they feel nauseous, dizzy, chest pain, or short of breath.
4. **COMPLETE ANTHROPOMETRICS & FUNCTIONAL ASSESSMENTS USING DATA COLLECTION FORM**

*(scan & upload to box or edit directly onto an electronic document and upload to box)*

- - - - -
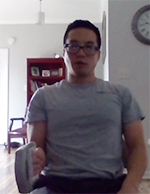

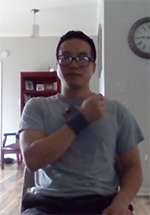

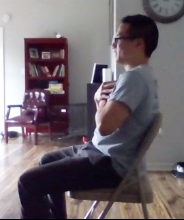
***Self-Reported Height & Weight***
        - ***Blood Pressure/ Heart Rate***

Equipment: a chair with arm, a HR monitor and BP cuff

Setup:

Camera view includes the front view of participant’s upper body.

Have the participant sit in a stationary chair or a wheelchair quietly for at least 5 minutes and use a HR monitor and BP cuff from the teleassesssment equipment package

- - - - - ***Grip Strength (90 °) Test***

Equipment: a chair with arm, a digital hand dynamometer

Setup:

Camera view includes the front view of participant’s upper body.

Have the participant sit in a stationary chair or a wheelchair and use a hand dynamometer from the teleassesssment equipment package

- - - - - ***Five Times Sit to Stand Test***

Equipment: a chair (you may use a chair with arms for safety but discourage participant from using if unnecessary)

Setup:

Camera view includes the side view of participant’s entire body (at least, shoulders, hip, and knees)

Have the participant sit in a stationary chair or a wheelchair

Three options for where to place a chair:


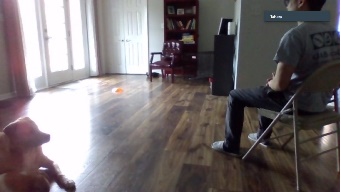
An open space, not supported against a mat or wall

An open space, support from a caregiver/or family member if necessary

Supported against a wall

- - - - - ***Timed Up & Go Test***

Equipment: a chair with arm, a mini disc cone, a 120-inch soft measuring tape

Setup:


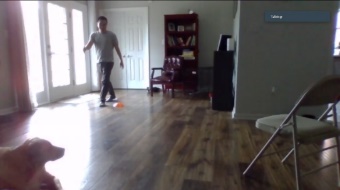

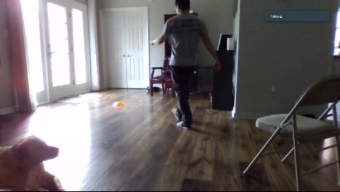
Camera view includes participant’s entire body throughout the test (to do so, the camera view should include the 3-meter walkway and the chair). You may ask participant to place the computer
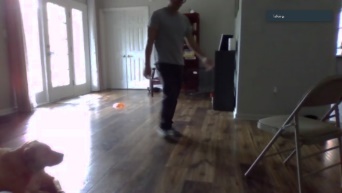

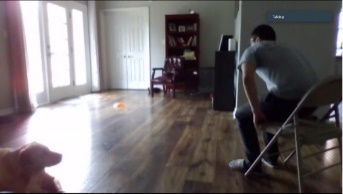
laptop on the floor or on the furniture to have better view.

Have the participant place a chair at the beginning of a 12-foot cleared space, free from obstacles or throw rugs. Next, have the participant lay down the measuring stick starting at the tip of their toes when sitting in a chair, then place the cone at the end of the measuring tape. Now remove the measuring tape and sit down in the chair.

Three options for where to place a chair:

An open space, not supported against a mat or wall

An open space, support from a caregiver/or family member if needed

Against wall, supported against a wall

- - - - -
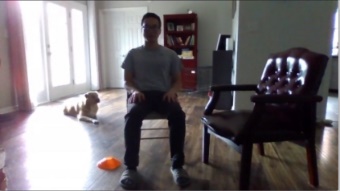
***Berg Balance Scale***

Equipment: two chairs (one with arms and one without), an 8-inch therapy step, a 36-inch adhesive measuring ruler, a minidisc cone

Setup:

Camera view includes the front view of participant’s entire body for all tasks. **First complete all tasks in the frontal view. After completing these tasks, perform the stand w/eyes closed and the stand with feet together task a second time with a side view.**

Three options for where to place a chair

An open space, not supported against a mat or wall

An open space, **support from a caregiver/or family member if possible**

Against wall, supported against a wall

**SITTING TO STANDING**


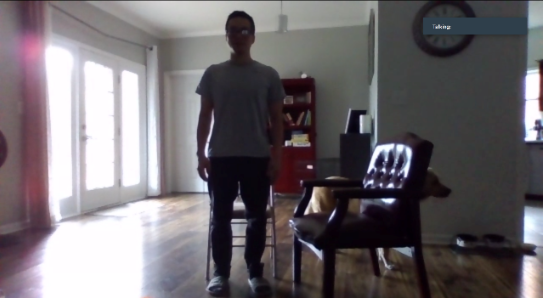


**STANDING UNSUPPORTED**


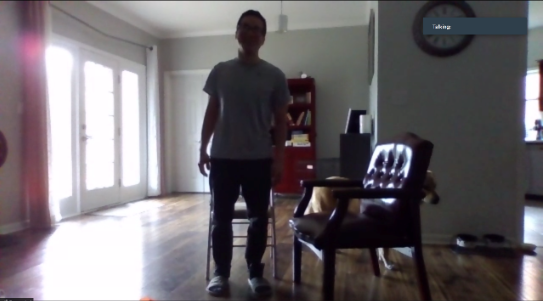


**SITTING W/ BACK UNSUPPORTED**


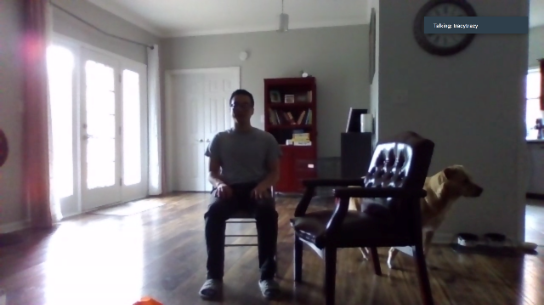


**STANDING TO SITTING**

**TRANSFERS**


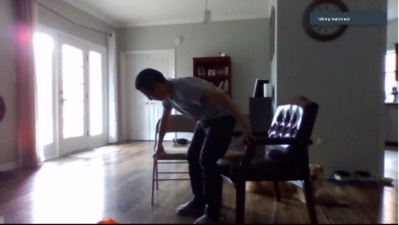

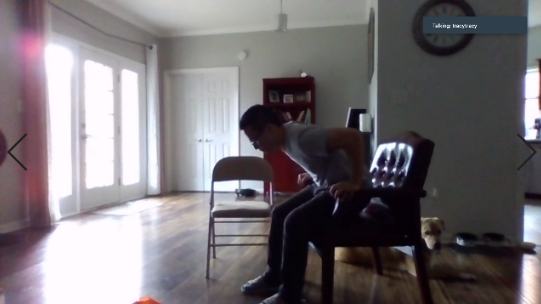


**STANDING UNSUPPORTED WITH EYES CLOSED**

**STANDING UNSUPPORTED WITH FEET TOGETHER**


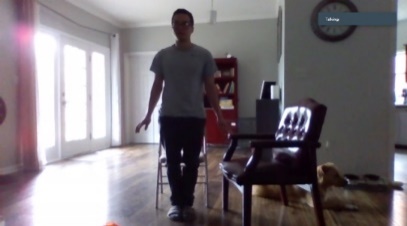


**REACHING FORWARD WITH OUTSTRETCHED ARM WHILE STANDING**

**
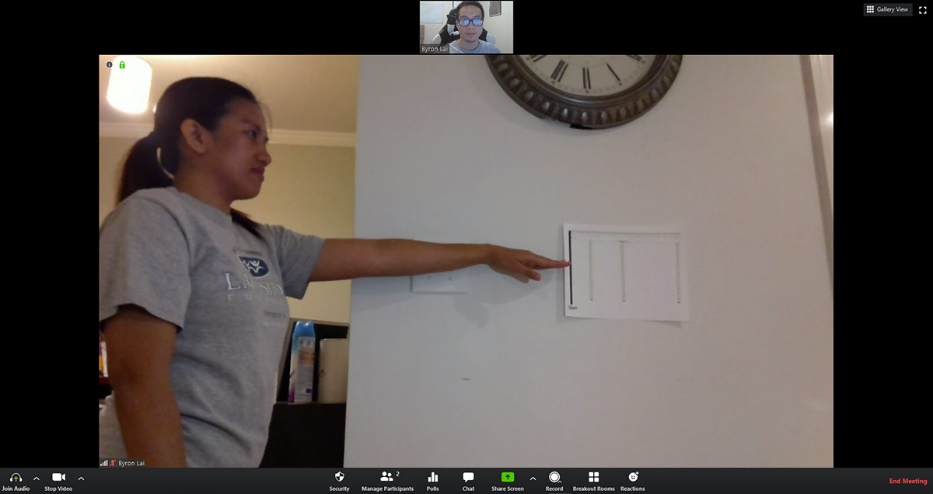
**

**(starting position)**

**PICK UP OBJECT FROM THE FLOOR**


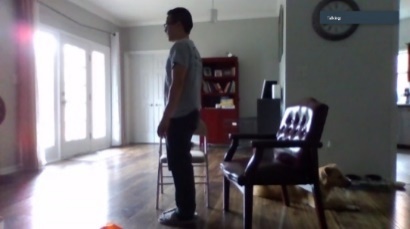


**TURNING TO LOOK BEHIND OVER LEFT AND RIGHT SHOULDERS WHILE STANDING**

**TURN 360 DEGREES**

**PLACING ALTERNATIVE FOOT ON STEP OR STOOL WHILE STANDING UNSUPPORTED**

**STANDING UNSUPPORTED ONE FOOT IN FRONT**


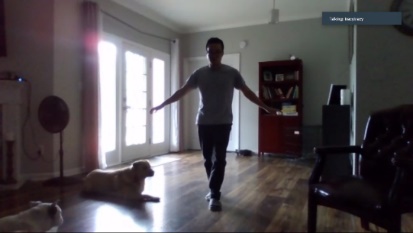


**STANDING ON ONE LEG**


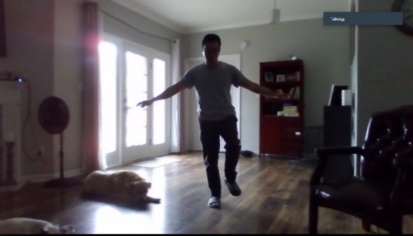


**REPEAT IN SIDE VIEW- STANDING UNSUPPORTED WITH EYES CLOSED**


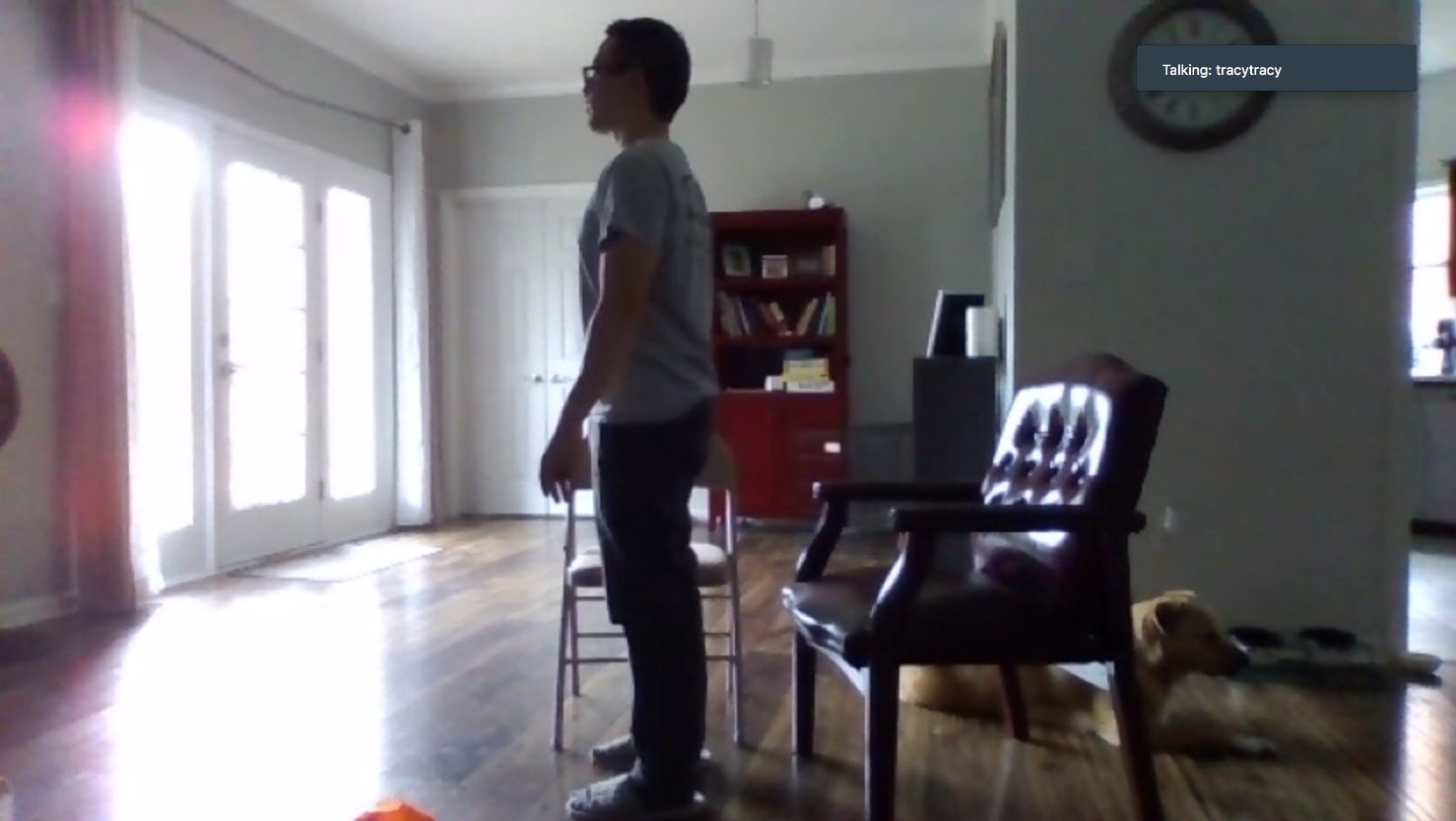


**REPEAT IN SIDE VIEW- STANDING UNSUPPORTED FEET TOGETHER**

1. **Remind participant to fill out questionnaires**
2. **Inform participants that they will be sent a gift card from research staff**
3. **Schedule follow-up visits**
4. **In the UAB Box activity tracking sheet, inform research staff that participant completed data collection and to send participant a gift card**
